# A young progenitor for the most common planetary systems in the Galaxy

**DOI:** 10.1038/s41586-025-09840-z

**Published:** 2026-01-07

**Authors:** John H. Livingston, Erik A. Petigura, Trevor J. David, Kento Masuda, James Owen, David Nesvorný, Konstantin Batygin, Jerome de Leon, Mayuko Mori, Kai Ikuta, Akihiko Fukui, Noriharu Watanabe, Jaume Orell Miquel, Felipe Murgas, Hannu Parviainen, Judith Korth, Florence Libotte, Néstor Abreu García, Pedro Pablo Meni Gallardo, Norio Narita, Enric Pallé, Motohide Tamura, Atsunori Yonehara, Andrew Ridden-Harper, Allyson Bieryla, Alessandro A. Trani, Eric E. Mamajek, David R. Ciardi, Varoujan Gorjian, Lynne A. Hillenbrand, Luisa M. Rebull, Elisabeth R. Newton, Andrew W. Mann, Andrew Vanderburg, Guðmundur Stefánsson, Suvrath Mahadevan, Caleb Cañas, Joe Ninan, Jesus Higuera, Kamen Todorov, Jean-Michel Désert, Lorenzo Pino

**Affiliations:** 1 Astrobiology Center, Mitaka, Japan; 2 National Astronomical Observatory of Japan, Mitaka, Japan; 3 Department of Astronomical Science, The Graduate University for Advanced Studies, Hayama, Japan; 4 Department of Physics & Astronomy, University of California Los Angeles, Los Angeles, CA USA; 5 Center for Computational Astrophysics, Flatiron Institute, New York, NY USA; 6 Department of Earth and Space Science, Osaka University, Osaka, Japan; 7 Astrophysics Group, Department of Physics, Imperial College London, London, UK; 8 Department of Earth, Planetary, and Space Sciences, UCLA, Los Angeles, CA USA; 9 Department of Space Studies, Southwest Research Institute, Boulder, CO USA; 10 Division of Geological and Planetary Sciences, Caltech, Pasadena, CA USA; 11 Komaba Institute for Science, University of Tokyo, Tokyo, Japan; 12 Instituto de Astrofísica de Canarias, La Laguna, Spain; 13 Observatoire astronomique de l’Université de Genève, Versoix, Switzerland; 14 Sabadell Astronomical Society, Sabadell, Spain; 15 Department of Astronomy, University of Tokyo, Tokyo, Japan; 16 Department of Astrophysics and Atmospheric Sciences, Kyoto Sangyo University, Kyoto, Japan; 17 Las Cumbres Observatory Global Telescope Network, Goleta, CA USA; 18 Center for Astrophysics, Harvard & Smithsonian, Cambridge, MA USA; 19 The Niels Bohr International Academy, Niels Bohr Institute, Copenhagen, Denmark; 20 National Institute for Nuclear Physics, Trieste, Italy; 21 Jet Propulsion Laboratory, California Institute of Technology, Pasadena, CA USA; 22 NASA Exoplanet Science Institute, IPAC, Pasadena, CA USA; 23 Department of Physics and Astronomy, Dartmouth College, Hanover, NH USA; 24 Department of Physics and Astronomy, UNC Chapel Hill, Chapel Hill, NC USA; 25 Department of Physics, Kavli Institute for Astrophysics and Space Science, Massachusetts Institute of Technology, Cambridge, MA USA; 26 Anton Pannekoek Institute for Astronomy, University of Amsterdam, Amsterdam, The Netherlands; 27 Department of Astronomy & Astrophysics, The Pennsylvania State University, University Park, PA USA; 28 Center for Exoplanets and Habitable Worlds, The Pennsylvania State University, University Park, PA USA; 29 NASA Goddard Space Flight Center, Greenbelt, MD USA; 30 Department of Astronomy and Astrophysics, Tata Institute of Fundamental Research, Mumbai, India; 31 NSF National Optical-Infrared Astronomy Research Laboratory, Tucson, AZ USA; 32 INAF-Osservatorio Astrofisico di Arcetri, Florence, Italy

**Keywords:** Exoplanets, Exoplanets, Atmospheric dynamics, Time-domain astronomy

## Abstract

The Galaxy’s most common known planetary systems have several Earth-to-Neptune-size planets in compact orbits^1^. At small orbital separations, larger planets are less common than their smaller counterparts by an order of magnitude. The young star V1298 Tau hosts one such compact planetary system, albeit with four planets that are uncommonly large (5 to 10 Earth radii)^2,3^. The planets form a chain of near-resonances that result in transit-timing variations of several hours. Here we present a multi-year campaign to characterize this system with transit-timing variations, a method insensitive to the intense magnetic activity of the star. Through targeted observations, we first resolved the previously unknown orbital period of the outermost planet. The full 9-year baseline from these and archival data then enabled robust determination of the masses and orbital parameters for all four planets. We find the planets have low, sub-Neptune masses and nearly circular orbits, implying a dynamically tranquil history. Their low masses and large radii indicate that the inner planets underwent a period of rapid cooling immediately after dispersal of the protoplanetary disk. Still, they are much less dense than mature planets of comparable size. We predict the planets will contract to 1.5–4.0 Earth radii and join the population of super-Earths and sub-Neptunes that nature produces in abundance.

## Abstract (web summary)

A nine-year transit-timing campaign has measured the extremely low masses and densities of four large planets orbiting the young star V1298 Tau, which are now predicted to contract and form a typical compact super-Earth and sub-Neptune system.

## Main

V1298 Tau is a young (10–30 Myr), approximately solar-mass star (1.10 ± 0.05 *M*_⊙_) in the Taurus star-forming region^2,4–8^. Observations by NASA’s Kepler space telescope in its extended K2 mission^9^ revealed transits of the star by four different planets, each larger than Neptune^2,3^. The V1298 Tau planets occupy a sparsely populated region of the observed exoplanet period versus radius plane. As a young system of large planets, it provides a crucial snapshot of planetary architecture just after formation, serving as the ‘missing link’ between protoplanetary disks and the mature systems found by Kepler^3^. Measuring their masses and orbits is, therefore, a key test of planet formation theories and allows us to witness early evolutionary processes, such as atmospheric mass loss, that sculpt planetary systems over billion year timescales.

**Fig. 1:**
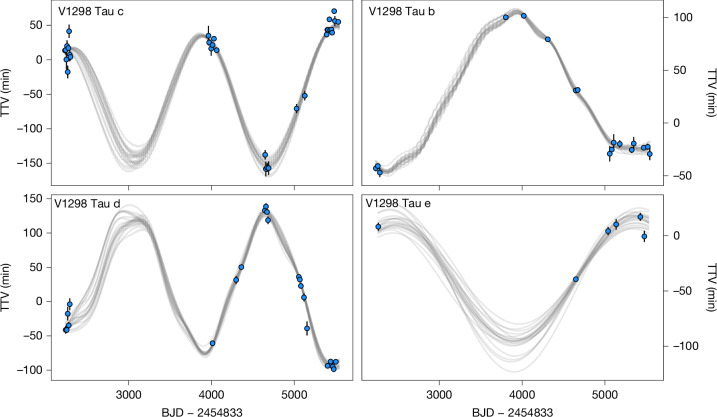
TTVs in the V1298 Tau system. Top left, points show the transit times of planet c measured against a reference linear ephemeris; error bars represent 1*σ* uncertainties. Grey curves show credible transit times drawn from the *N*-body models described in the text. Bottom left, same as above but for planet d. The interactions between c and d are nearly sinusoidal and anticorrelated. Top and bottom right, the same but for planets b and e. The TTVs of b and e are also sinusoidal and anticorrelated.

Between 2019 and 2024, we observed 43 other transits of all four planets using both space- and ground-based telescopes. This campaign successfully recovered the previously lost outermost planet, V1298 Tau e, and resolved a long-standing period ambiguity^10^ (Methods). We performed homogeneous and self-consistent modelling of all transit data from 2015 to 2024. After determining the transit shape parameters, we fit the midpoint of each transit individually (Methods). The transit-timing variations (TTVs) are shown in Fig. 1. All planets exhibit significant TTVs with amplitudes ranging from  approximately 50 min to 100 min. Moreover, the TTVs of the c–d pair are anticorrelated, as are those of the b–e pair. This indicates that the c–d and b–e interactions dominate over other pairwise interactions.

Previous works have developed analytic models of TTVs applicable to certain orbital configurations^11,12^. Other works have developed *N*-body TTV models based on a dynamical integration of the star–planet system subject to Newtonian gravity (for example, refs. ^13–15^). Analytic models are generally faster to evaluate, have fewer free parameters and offer a clearer connection between the system properties and the TTV waveform. *N*-body models, with more free parameters, are slower to evaluate but can completely describe any star–planet system.

Most TTV studies in the literature treat planets from the 4-year Kepler mission. These studies had the benefit of near-continuous sampling over a full TTV period. The sparse sampling of our dataset presents different challenges. We, therefore, used analytic models to build our intuition of the system dynamics before undertaking a full *N*-body analysis. The nature of TTV interactions depends on the proximity to resonance *Δ*, defined as $\varDelta =\frac{{P}_{2}}{{P}_{1}}\frac{j-1}{j}-1$, where *P*_1_ and *P*_2_ are the orbital periods of the inner and outer planets, respectively, and *j* is a positive integer defining the resonance, with smaller *Δ* associated with larger TTVs. In this system, *Δ*_cd_ = 0.2%, *Δ*_db_ = −2.7% and *Δ*_be_ = 0.8%. Given the strength of the c–d and b–e interactions over d–b interactions, we used analytic models to treat the c–d and b–e interactions separately. The analytic models indicated low masses and low eccentricities (Methods).

Guided by our analytic results, we then performed a full *N*-body dynamical fit to the transit times to derive a final, robust set of planet parameters. This model accounts for all gravitational interactions in the system simultaneously, including subtle, higher-order TTVs that can help break degeneracies inherent in our analytic models. Details of our *N*-body model, Bayesian statistical framework and Markov chain Monte Carlo sampling are provided in Methods.

Figure 1 shows a selection of credible models drawn from our posterior samples, along with the timing data. The models fit the data well, with increased scatter where observations are sparse. The credible range of each planetary parameter is listed in Table 1. We find masses *M*_c_ = 4.7 ± 0.6 *M*_⊕_, *M*_d_ = 6.0 ± 0.7 *M*_⊕_, *M*_b_ = 13.1 ± 5.3 *M*_⊕_ and *M*_e_ = 15.3 ± 4.2 *M*_⊕_ (the uncertainties correspond to the 68% highest density intervals of the marginal posteriors). In addition, the planetary eccentricities are all  less than about 1%. A detailed dynamical analysis (Methods) confirms that this solution corresponds to a long-term stable and non-resonant orbital architecture. The *N*-body results are consistent with the analytic results at 2*σ* or better with smaller uncertainties. The *N*-body model is a more complete description of the planetary dynamics than our analytic models and includes effects like synodic chopping^16^ and d–b interactions. It exhibits root mean square (r.m.s.) values of 4–11 min, consistent with our analytic models. Henceforth, we adopt and interpret the *N*-body results. The masses of planets b and e have substantial fractional uncertainties but are distinct from zero; they are larger than 4.8 *M*_⊕_ and 7.8 *M*_⊕_ to 95% confidence. The broad uncertainties stem from the well-known mass versus eccentricity degeneracy^11^. Extremely low masses and high eccentricities would produce b–d interactions that are inconsistent with the data.

**Table 1:** Planetary parameters

Parameter	Unit	c	d	b	e
*M*_p_	*M*_⊕_	4.7 ± 0.6	6.0 ± 0.7	13.1 ± 5.3	15.3 ± 4.2
*R*_p_	*R*_⊕_	5.08 ± 0.37	6.53 ± 0.42	9.41 ± 0.57	10.17 ± 0.75
*ρ*	g cm^−3^	0.20 ± 0.05	0.12 ± 0.03	0.09 ± 0.04	0.08 ± 0.03
*e*	%	<0.94	<0.87	0.79 ± 0.41	<1.24
*P*	days	8.249164(3)	12.401394(9)	24.140006(17)	48.677714(53)
*a*	au	0.0824 ± 0.0012	0.1081 ± 0.0016	0.1685 ± 0.0025	0.2689 ± 0.0040
*T*_eq_	K	953 ± 36	831 ± 31	666 ± 25	527 ± 20

Parameter definitions: *M*_p_, planetary mass; *R*_p_, planetary radius; *ρ*, bulk density; *e*, orbital eccentricity; *P*, orbital period; *a*, semi-major axis; and *T*_eq_, equilibrium temperature. Uncertainties denote the 68% credible interval and upper limits are 1*σ*. *T*_eq_ values assume a stellar effective temperature value of *T*_eff_ = 4,970 ± 120 K (ref. ^2^) and zero Bond albedo. Planet-to-star mass and radius ratios are converted to planetary masses and radii using a stellar mass value of *M*_⋆_ = 1.10 ± 0.05 *M*_⊙_ and a stellar radius value of *R*_⋆_ = 1.32 ± 0.05 *R*_⊙_ (ref. ^2^). Eccentricities are given in percent.

The combination of low planet masses with the youth of the host star makes Doppler mass measurements challenging. The expected semi-amplitudes of the radial velocity are  approximately 1–2 m s^−1^, which are two orders of magnitude smaller than the stellar activity signal. For comparison, the measured r.m.s. values of the radial velocity in existing datasets for V1298 Tau are 260 m s^−1^, 197 m s^−1^ and 195 m s^−1^ for HARPS-N, CARMENES VIS and CARMENES NIR, respectively^7^. The challenge of this high stellar activity has been a central theme in recent studies of the system^17–20^. Reference ^7^ simultaneously modelled planetary and activity radial velocity variations, reporting masses of 203 ± 60 *M*_⊕_ and 367 ± 95 *M*_⊕_ for planets b and e, an order of magnitude larger than our TTV results. However, ref. ^21^ found that the planet-activity model is biased towards over-predicting planet masses when stellar activity dominates. Given these challenges and the risk of systematic bias, a TTV-only analysis provides, at present, the most robust and unbiased mass constraints for this system. Notably, our dynamical mass for planet b is consistent with independent atmospheric constraints. A recent analysis of transmission spectra captured by the James Webb Space Telescope by ref. ^22^ inferred a mass from the atmospheric scale height that is in excellent agreement with our TTV result. That two independent methods—one based on gravitational dynamics and the other on atmospheric structure—yield such consistent results provides a powerful validation of our measurement.

**Fig. 2:**
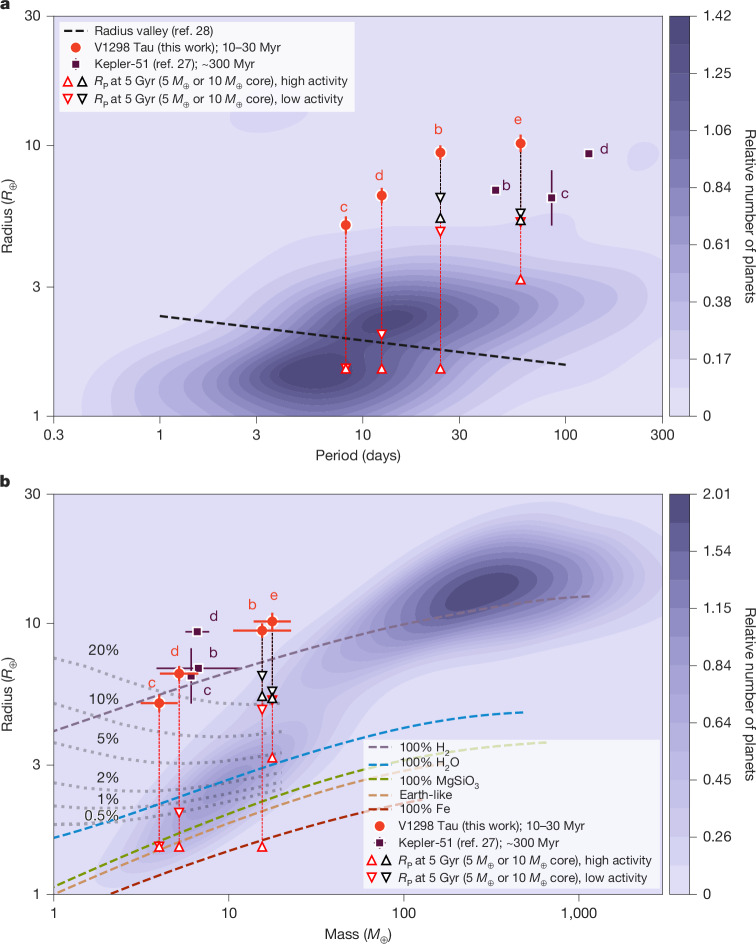
Planetary radius versus orbital period and planetary mass. **a**,**b**, Planetary radius versus orbital period (**a**) and planetary radius versus planetary mass (**b**) for the V1298 Tau system (red filled circles); error bars represent 1*σ* uncertainties. The low-density planets of the Kepler-51 system are shown for comparison (purple squares), along with kernel density estimates of the distributions of well-characterized exoplanets (shaded contours), drawn from the NASA Exoplanet Archive (*n* = 624 planets with mass and radius uncertainties less than 20%, *P* < 150 days and host *T*_eff_ = 4,500–6,500 K to exclude M dwarfs). The parameters of the Kepler-51 planets were sourced from the ‘outside 2:1’ solution in Table 6 of ref. ^27^. Theoretical radius evolution tracks from ref. ^29^ are shown as vertical dashed lines. The terminal radii at 5 Gyr from that work are shown as open triangles. The colour indicates the assumed core mass (red for 5 *M*_⊕_ and black for 10 *M*_⊕_). The orientation represents the stellar extreme-ultraviolet activity level (upwards for high activity and downwards for low activity). The black dashed line in **a** depicts the observed location of the radius valley^28^. Theoretical mass–radius relations for different planet compositions from ref. ^31^ are shown in **b** as dashed lines. Grey dotted lines indicate theoretical mass–radius relations for Earth-like cores with H/He envelopes with various mass fractions from ref. ^30^, calculated for an age of 100 Myr and an insolation of 10 *F*_⊕_.

The planetary densities that we measured in the V1298 Tau system are among the lowest exoplanet densities recorded. The only known multi-planet system exhibiting comparably low densities is, perhaps not coincidentally, the young (approximately 300 Myr) transiting system Kepler-51, for which mass measurements were also made through TTVs^23–27^, although V1298 Tau is significantly younger and more compact. Figure 2 places the V1298 Tau planetary system in the context of the broader, mature exoplanet population. Figure 2a shows these young planets positioned above the radius gap^28^. To trace their future evolution, we overplot the ‘fluffy’ planet scenarios from ref. ^29^, which are the most relevant analogues. These models bracket a range of possibilities by assuming two different core masses (5 *M*_⊕_ and 10 *M*_⊕_) and two stellar extreme-ultraviolet activity levels that result in different degrees of atmospheric mass loss. The 5 *M*_⊕_ scenario is a particularly strong analogue, as our own interior structure modelling (Methods) constrains the core masses of planets c and d to be 4–6 *M*_⊕_ (1*σ*). The resulting tracks indicate that some planets will contract across the gap to become super-Earths, whereas others will become sub-Neptunes, thus directly tracing the formation of the bimodal radius distribution observed by Kepler. Figure 2b reveals substantial H/He envelopes^30^, although their final evolved states may have densities that are degenerate with water worlds^31^.

The low masses and densities of the V1298 Tau planets have significant ramifications for planet formation theory. Theoretical modelling indicated that planet c (*M*_c_ = 4.7 ± 0.6 *M*_⊕_) was one of the best targets for constraining its formation history: a mass higher than 10 *M*_⊕_ would be consistent with standard core-accretion models, whereas a mass lower than 6 *M*_⊕_ would require a ‘boil-off’ phase during protoplanetary disk dispersal^32^. Such a phase occurs when the pressure support of the disk is removed swiftly, triggering profuse atmospheric mass loss through a Parker wind and rapid cooling, leaving behind an envelope with lower entropy and a longer Kelvin–Helmholtz timescale compared with predictions from standard core-accretion models^33,34^.

To explore the possible formation channels for the V1298 Tau planets, we modelled the planets as two-layer objects consisting of an Earth composition rocky core ensheathed in a H/He envelope. The initial envelope entropy is parameterized by its Kelvin–Helmholtz contraction timescale. We ran a dense grid of models spanning core mass, initial envelope mass fraction and initial envelope entropy at the location of each planet in the system and evolved them to the current age of the system.

**Fig. 3:**
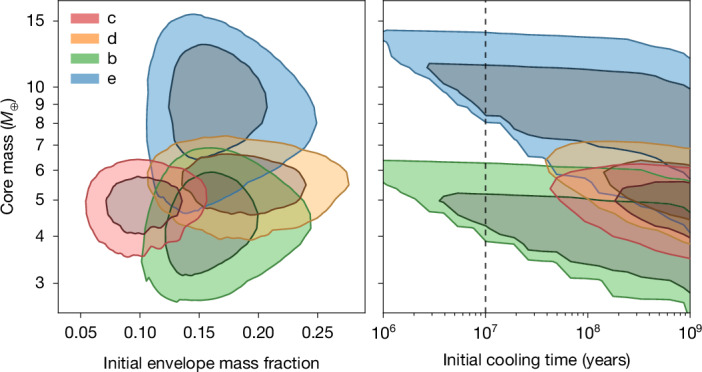
Posterior distributions for the initial properties of the V1298 Tau planets. The posteriors were derived by applying the planetary evolution and mass loss framework of ref. ^32^ to our measured masses and radii for planets c (red), d (orange), b (green) and e (blue). Left, initial envelope mass fraction versus core mass. Right, initial Kelvin–Helmholtz cooling timescale versus core mass. Contours show the 1*σ* and 2*σ* credible regions. (Note that the jagged appearance of some contours is a numerical artefact of the discrete core mass grid used in our analysis; see Methods for more details). The vertical dotted line in the right panel at 10 Myr marks the approximate upper limit for standard high-entropy formation models. These models are strongly disfavoured for the inner planets c and d, whereas for the less-irradiated outer planets b and e, the method lacks the statistical power to distinguish between high- and low-entropy scenarios.

Figure 3 shows posterior distributions for the initial properties of all four planets, providing a deeper insight into the system architecture. The right panel confirms that the inner planets c and d require low-entropy initial states (much greater than 30 Myr Kelvin–Helmholtz cooling times), whereas the less-irradiated outer planets b and e remain unconstrained. The left panel, however, reveals a notable uniformity: all four planets are consistent with having similar core masses (approximately 4–6 *M*_⊕_) and initial envelope mass fractions (approximately 0.1–0.2). This indicates that the system is an exemplar of the ‘peas in a pod’ phenomenon at formation^35^, implying its present-day size diversity is a transitory phase driven by different levels of photoevaporation.

**Extended Data Fig. 1:**
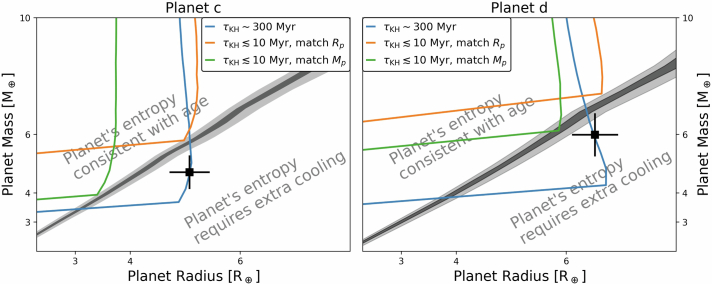
Mass-radius diagram for V1298 Tau c (left) and d (right) at the system’s age of 23 Myr. The gray shaded region marks the boundary of parameter space accessible by standard, high-entropy formation models (initial Kelvin-Helmholtz cooling timescale *τ*_KH_ ≲ 10 Myr), with the 1-*σ* and 2-*σ* uncertainty on its location shown in dark and light gray. Illustrative model tracks are shown for comparison: the green and orange lines demonstrate that high-entropy models fail to simultaneously match the measured mass and radius, while the blue line shows a successful low-entropy model (*τ*_KH_ ~ 300 Myr) consistent with the data. The positions of planets c and d firmly in the lower-right region provide direct evidence that their formation required a low-entropy initial state, such as that produced by a ‘boil-off’ phase.

Extended Data Fig. 1 shows that the measured masses and radii of planets c and d lie outside the region of parameter space accessible to standard, high-entropy core-accretion models. As the illustrative tracks in the figure demonstrate, only lower-entropy (boil-off) models can simultaneously satisfy both the mass and radius constraints after accounting for 23 Myr of evolution and mass loss. During boil-off, the planetary envelope becomes over-pressurized and expands hydrodynamically, carrying away significant internal energy and leaving behind a cooler interior^33,34,36^. Although our measurements support boil-off for the inner planets, recent atmospheric retrievals indicating a high internal temperature for planet b^37^ present a possible tension that merits further investigation.

Theoretical modelling of the system under the influence of extreme-ultraviolet- and X-ray-driven photoevaporation indicates that these planets will continue to lose mass over the next 100 million years^8,29^, even though they have already experienced significant atmospheric loss. For our measured masses, standard evolutionary models predict that all the planets will retain a small fraction of their initial atmospheres, although the inner two could become stripped, depending on the future spin evolution of the star^29^. Interestingly, observational searches for continuing atmospheric escape have so far yielded inconclusive results^5,38–40^, possibly because strong stellar winds act to suppress planetary outflows^41–43^.

## Methods

### Transit observations and analysis

We analysed a heterogeneous dataset of light curves from space- and ground-based telescopes (Supplementary Table 1) to measure transit times for the ultimate purpose of modelling TTVs. We used PyMC3^44^, exoplanet (https://docs.exoplanet.codes/en/stable/)^45^ and starry^46^ to fit the light curve, incorporating tailored models for correlated noise and instrumental systematics appropriate for each dataset.

Our analysis of the K2 and Transiting Exoplanet Survey Satellite (TESS) light curves involved two distinct approaches with different noise models. For the joint analysis of all transits in both light curves (described below), we modelled stellar variability as a Gaussian process^47^. By contrast, for measuring individual transit times (see below), a third-order basis spline was sufficient to model the local correlated noise.

To account for systematics in the Spitzer data, we used pixel-level decorrelation (PLD)^48^, which uses a linear model with a design matrix formed by the PLD basis vectors (see below for more details). For the ground-based datasets, we included a linear model with a design matrix formed by airmass, pixel centroids, and the pixel response function peak and width covariates, when available.

The limb-darkening coefficients were calculated using stellar parameters from ref. ^2^ by interpolation of the parameters tabulated by refs. ^49,50^. These were fixed for individual transit fits but sampled with uninformative priors in the joint K2 and TESS analysis described below.

We used Broyden–Fletcher–Goldfarb–Shanno optimization^51^ as implemented in scipy.optimize for initial parameter estimates, followed by posterior sampling with the No-U-Turn Sampler^52^, an efficient gradient-based Hamiltonian Monte Carlo sampler implemented in PyMC3. The chains were well mixed (Gelman–Rubin statistic  less than about  1.01) with negligible sampling error.

We first performed a joint fit of K2 and TESS data assuming a linear ephemeris (see below). We then measured all individual transit times uniformly using Gaussian priors from the joint fit for *R*_p_/*R*_⋆_, *b* (the transit impact parameter), and *T*_14_ (the total transit duration), and uniform priors for *T*_c_ (the transit centre time) centred on predicted times. We verified that *T*_c_ posteriors were Gaussian and isolated well from prior edges.

In all individual transit fits, we assumed Gaussian independent and identically distributed noise and included a jitter parameter *σ*_jit_ to account for underestimated photometric uncertainties. The log-likelihood was, thus,

$$\text{ln}{\mathcal{L}}=-\frac{1}{2}\text{ln}| \varSigma | -\frac{1}{2}{{\bf{r}}}^{{\rm{T}}}{\varSigma }^{-1}{\bf{r}}+{\rm{const.}},$$

where *Σ* is the diagonal covariance matrix with entries equal to the total variance (that is, the *i*th entry is ${\sigma }_{{\rm{tot,}}i}^{2}={\sigma }_{{\rm{obs,}}i}^{2}+{\sigma }_{{\rm{jit}}}^{2}$, where *σ*_obs,*i*_ is the observational uncertainty of the *i*th data point), and **r** is the residual vector (${\bf{r}}=[{\widehat{y}}_{1}-{y}_{1},{\widehat{y}}_{2}-{y}_{2},\ldots ,{\widehat{y}}_{n}-{y}_{n}]$, where $\widehat{y}$ is the model and *y* is the data consisting of *n* measurements). When a given transit event was observed by several telescopes (for example, at Las Cumbres Observatory (LCO))^53^, or several band-passes from the same instrument (for example, from MuSCAT3)^54^, we jointly fitted all light curves covering the same event.

We obtained ground-based follow-up transit observations from a variety of facilities spanning several observing seasons. Early in the project, observations were distributed diversely among a half-dozen telescopes, but later we focused almost exclusively on the LCO telescope network, which enabled both the acquisition of data and its analysis to be conducted more uniformly. The individual dates, facilities, band-passes and exposure times of these observations are listed in chronological order in Supplementary Table 1. The measured transit times are provided in Supplementary Table 2.

#### Joint analysis of the K2 and TESS light curves

V1298 Tau (EPIC 210818897) was observed between 7 February and 23 April 2015 during campaign 4 of the K2 mission^9^. We analysed the K2 light curve produced by the EVEREST pipeline (https://github.com/rodluger/everest)^55,56^, which is available at the Mikulski Archive for Space Telescopes (MAST) (https://archive.stsci.edu/hlsp/everest).

V1298 Tau (TIC 15756231) was observed at 2-min cadence in Sectors 43 and 44 (16 September to 6 November 2021) of the TESS mission^57^ as part of the Director’s Discretionary Time (DDT) programme 036 (PI T. David).

**Extended Data Fig. 2:**
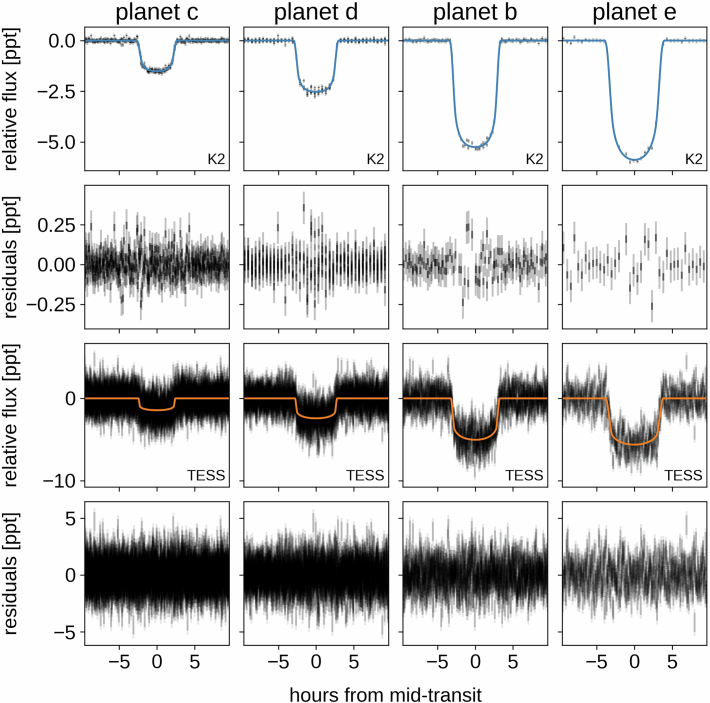
*K2* and *TESS* data with models from the joint fit. The top two rows show the variability-corrected *K2* light curves centered on the transit of each planet with the best-fit transit model over-plotted, with the residuals from the fit shown below; dark error bars show the measured photometric uncertainties, while the lighter error bars denote the error bars including the jitter value from the fit. The bottom two rows show the same, but for the *TESS* data.

We conducted a joint fit to the K2 and TESS light curves assuming a linear ephemeris, using a Gaussian process to account for correlated noise arising from a combination of stellar variability and instrumental systematics (based on the tutorial available at https://gallery.exoplanet.codes/tutorials/lc-multi/). We used a simple-harmonic-oscillator covariance function with a power spectral density given by:

$$S(\omega )=\sqrt{\frac{2}{{\rm{\pi }}}}\frac{{S}_{0}{\omega }_{0}^{4}}{{({\omega }^{2}-{\omega }_{0}^{2})}^{2}+{\omega }_{0}^{2}{\omega }^{2}/{Q}^{2}},$$

where *ω* is the angular frequency, *ω*_0_ is the undamped angular frequency of the oscillator and *S*_0_ is a scale factor that sets the amplitude of the variability. This was re-parameterized by the undamped period of the oscillator *ρ* (defined as *ρ* = 2π/*ω*_0_), the standard deviation of the process *σ* (defined as $\sigma =\sqrt{{S}_{0}{\omega }_{0}Q}$) and the quality factor *Q* (fixed to 1/3). Like our model for individual transits, we included a photometric jitter term (*σ*_jit_), the square of which was added to the diagonal of the covariance matrix. The likelihood was, thus, identical to that shown for individual transits above, but the covariance matrix contained non-zero off-diagonal elements determined by the covariance function. The results of this fit are shown in Extended Data Fig. 2 and the posteriors are summarized in Extended Data Table 1.

#### Individual K2 and TESS transits

To create a uniform transit-timing dataset, we analysed individual transits from the long-baseline K2 and TESS light curves in the same manner as our short-duration follow-up observations. We constructed individual datasets from windows of three times the transit duration centred on each transit event. When there were overlapping transits, we used the longest transit duration and centred the window on the approximate midpoint of the dimming event. Unlike follow-up datasets, which are often partial transits, stellar variability is typically nonlinear on the timescale of these datasets. To account for this, we included a third-order basis spline with five evenly spaced knots. The transits of V1298 Tau c on 10 and 26 October 2021 ut resulted in poor-quality fits, probably due to the presence of short-timescale red noise close to ingress or egress or a low signal-to-noise ratio; as the timing posteriors from these fits were highly non-Gaussian, we discarded them from subsequent analyses.

#### Spitzer

**Extended Data Fig. 3:**
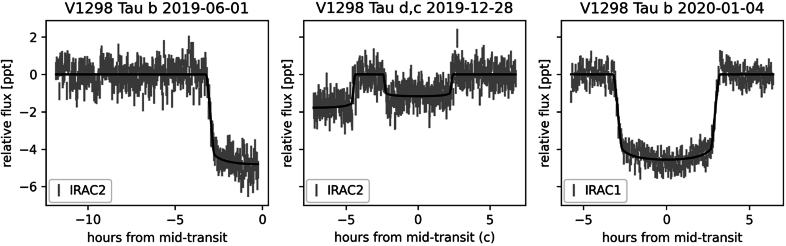
*Spitzer* observations used in this work with best-fit transit models. The photometry has been corrected for stellar variability and systematics.

We used the ephemeris derived from the K2 observations^2^ to predict transits of V1298 Tau b within Spitzer visibility windows in 2019. Subsequently, we did the same for V1298 Tau c,d using the ephemerides from ref. ^3^. Another transit of V1298 Tau b was scheduled in early 2020 using an updated ephemeris based on ref. ^2^ and the first Spitzer observation of that planet. The Spitzer data and best-fitting transit models are shown in Extended Data Fig. 3.

The first epoch of Spitzer observations of V1298 Tau were acquired as part of the DDT programme 14227 (PI E. Mamajek) and executed on 1 June 2019 ut. The second epoch of Spitzer observations were acquired as part of the target of opportunity programme 14011 (PI E. Newton) and executed on 28 December 2019 ut. In both epochs, data were acquired with channel 2 of the infrared array camera (IRAC) onboard Spitzer (with effective wavelength *λ*_eff_ = 4.5 μm) in the subarray mode using 2-s exposures. A third epoch of Spitzer observations were acquired in IRAC channel 1 (*λ*_eff_ = 3.6 μm) as part of DDT 14276 (PI K. Todorov) and executed on 4 January 2020 ut.

We extracted photometry following ref. ^58^ and modelled the instrumental systematics using PLD, which combines normalized pixel light curves as basis vectors in a linear model:

$${M}_{{\rm{PLD}}}^{t}({\boldsymbol{\alpha }})=\frac{{\sum }_{i=1}^{9}{c}_{i}{P}_{i}^{t}}{{\sum }_{i=1}^{9}{P}_{i}^{t}},$$

where *P*_*i*_ is the *i*th pixel light curve, the superscript *t* denotes the value at a specific time step, and **α** = {*c*_1_, …, *c*_9_} are the coefficients of the PLD basis vectors. The first epoch, which captured a partial transit of V1298 Tau b over approximately 11.5 h, was fitted well by including a linear trend in addition to PLD. The second epoch, which contained transits of both planets c and d over an approximately 14-h baseline, exhibited significant nonlinear variability that required the inclusion of a basis spline. Similarly, the third epoch, containing a full transit of planet b over approximately 12.5 h, also warranted the inclusion of a basis spline; although IRAC1 systematics are typically larger than those of IRAC2, PLD performed well and we attribute this to stellar variability. We validated our approach of selecting the baseline model by inspecting the fit residuals for the longest and most complex observation (the second epoch). A quantitative comparison confirmed that a basis spline was strongly preferred over a simple linear trend by the Bayesian information criterion^59^.

#### Ground-based observations

Most of our follow-up transit observations were obtained from 2020 to 2024 using LCO. We primarily used the Sinistro^53^ and MuSCAT3 instruments on the 1-m and 2-m telescopes, respectively.

In addition to LCO, we used data from a variety of other facilities, including Apache Point Observatory (APO)/Astrophysical Research Consortium Telescope Imaging Camera (ARCTIC)^60^, Fred Lawrence Whipple Observatory/KeplerCam^61^, WIYN/half degree imager^62^, Three-hundred MilliMeter Telescope^63^, MuSCAT^64^, MuSCAT2^65^ and Araki/ADLER. Data were obtained using a variety of filters and reduced using standard pipelines and methods^66–71^. See Supplementary Information for more details.

#### Recovering planet e

The outermost planet, V1298 Tau e, transited only once during the K2 mission. TESS recovered transits of all four planets, including a second transit of planet e^10^. It was not clear how many transits occurred between the K2 and TESS observations given the 6.5-year gap between the two campaigns. Thus, a discrete comb of periods was allowed, such that *P* = Δ*t*/*n*, where Δ*t* is the measured time between transit midpoints and the integer $n=1,2,3,\ldots ,{n}_{\max }$. The upper bound on *n*, and thus, the lower bound on a period of 42.7 days, was provided by the absence of other transits by planet e within the K2 and TESS time series^10^.

**Extended Data Fig. 4:**
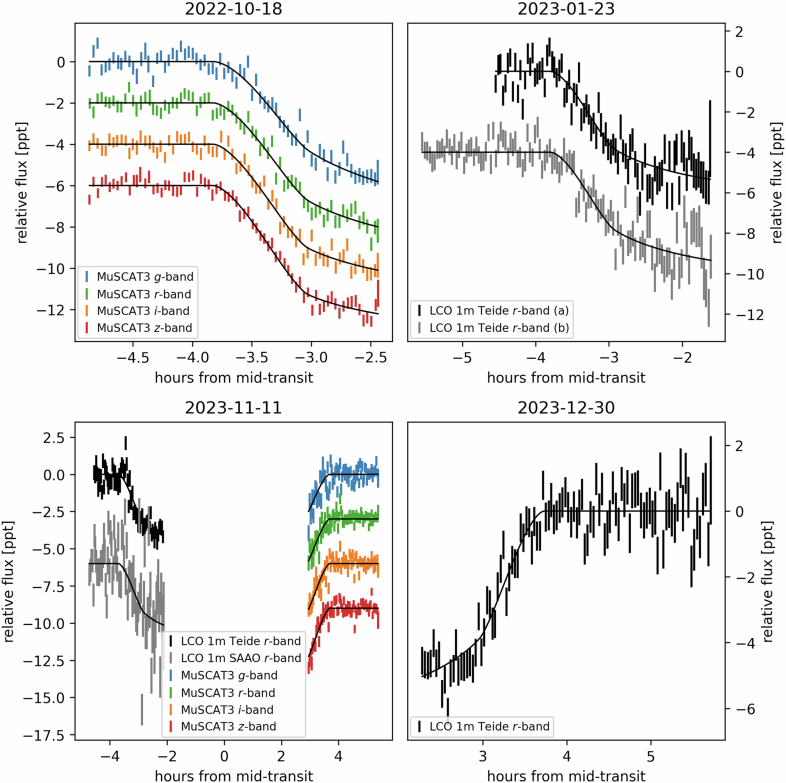
Recovery of V1298 Tau e from ground-based transit observations. The top left panel shows the partial transit recovered on 2022 October 18, which resolved the period ambiguity. The remaining panels show additional follow-up observations.

By the summer of 2022, a preliminary version of our timing dataset had revealed large TTVs of planet b that we assumed were dominated by interactions with planet e. We ran a suite of TTV models at each of the possible *P*_e_ between 42.7 days and 120 days. Few trial periods yielded good fits to the timing dataset, and dynamical simulations revealed that only a fraction of those were stable over ${\mathcal{O}}(1{0}^{6})$ years. One of the stable solutions with *P*_e_ = 48.7 days corresponded to a near 2:1 commensurability for the b–e pair, a common configuration among the Kepler planets exhibiting large and detectable TTVs. With this prediction, we recovered a partial transit of planet e from the ground on 18 October 2022. LCO datasets used to recover planet e and confirm its orbital period are shown in Extended Data Fig. 4.

#### Datasets containing flares

**Extended Data Fig. 5:**
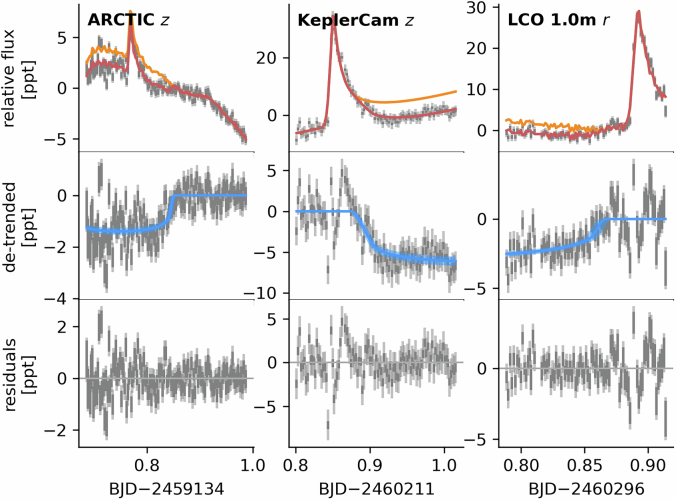
Datasets containing flares: a transit of planet c from ARCTIC (left); a transit of planet e from KeplerCam (middle); a transit of planet d from LCO (right). The residuals from the fit to the KeplerCam data have a feature that may be due to a smaller, secondary flare.

Several observations were affected by stellar flares and were excluded from our TTV analyses to avoid potential timing measurement biases. These datasets were modelled using our standard approach, augmented with a parametric flare model^72^ (Extended Data Fig. 5). Significant flares were observed in ARCTIC data (12 October 2020; see also ref. ^38^), KeplerCam data (24 September 2023) and LCO data (18 December 2023), with amplitudes ranging from 6 parts per thousand (ppt) to 42 ppt and timescales of 14 min to 21 min. The parameters of these flares are detailed in the Supplementary Information, and they may prove valuable for future studies of the activity of V1298 Tau.

### Mass constraints from analytic TTV modelling

To build intuition for the system dynamics, we first performed a preliminary analysis using analytic models of TTVs. Based on the foundational analytic frameworks for TTVs^11,12,73^, we determined that the system dynamics can be effectively decoupled into two pairs of planets: c–d and b–e.

**Extended Data Fig. 6:**
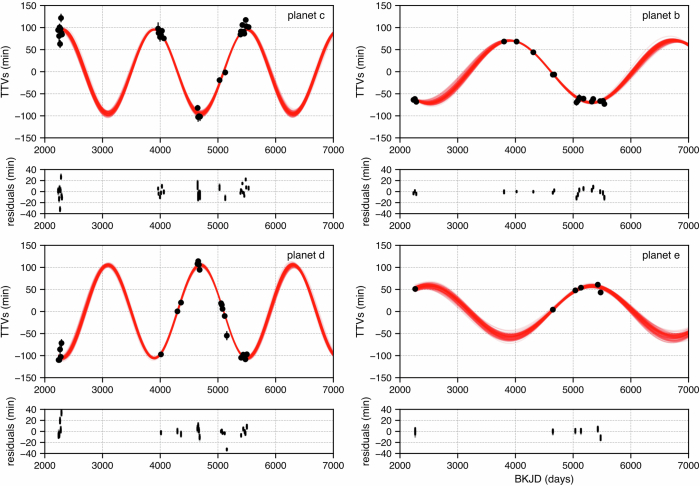
Multi-harmonic model of V1298 Tau TTVs described in Methods. The top left panel shows the measured deviations of planet c’s transit times from the best-fitting linear ephemeris. The red lines show draws from credible models. The second panel shows the difference between measured times and the model predictions. Other panels show the same quantities for planets d, b and e. The TTVs of planets c and d are dominated by interactions between the two planets. They are well-described by a sinusoid with an amplitude of ~100 min and a period of *P*_cd_ = 1604 ± 12 days. The TTVs of planets b and e are dominated by interactions between the two planets. Here, the TTVs are well-described by a sinusoid with an amplitude of ~50 min and a period of 2852 ± 50 days.

To quantify the TTV behaviour, we fitted a multi-harmonic sinusoidal model to the transit time series (see Supplementary Information for the model equations). We explored the posterior distributions of the 16 model parameters using a Markov chain Monte Carlo sampler, like approaches used by other public TTV analysis codes^74,75^. The posteriors of these parameters are listed in Supplementary Table 3, and the model fits are shown in Extended Data Fig. 6.

The results for the c–d pair are consistent with the planets being in a near-resonant regime. The TTVs are described well by a single sinusoid with a period *P*_cd_ = 1,604 ± 12 days and an r.m.s. of the residuals of only 11 min. This sinusoidal signal is dominated by variations in the planetary mean longitudes (*λ*), characteristic of systems very close to resonance. The ratio of the TTV amplitudes is sensitive to the planetary mass ratio, indicating nearly equal masses (*M*_d_/*M*_c_ ≈ 1.2). From the full fit, we derived preliminary masses ${M}_{{\rm{c}}}\approx {2.7}_{-0.8}^{+1.7}$ *M*_⊕_ and ${M}_{{\rm{d}}}\approx {3.2}_{-1.0}^{+2.1}$ *M*_⊕_.

By contrast, the b–e pair is described well by a simpler, linear TTV model, as it is further from resonance. The TTVs arising from variations in mean longitude and eccentricity have the same frequency in this regime. In this case, a well-known degeneracy exists between the planet masses and their orbital eccentricities^16,76^, leading to broader initial constraints of ${M}_{{\rm{b}}}=3{1}_{-17}^{+14}$ *M*_⊕_ and ${M}_{{\rm{e}}}=2{4}_{-8}^{+4}$ *M*_⊕_. A full theoretical treatment and a discussion of strategies for breaking the mass–eccentricity degeneracy, such as measuring secondary eclipse times^77^, can be found in the Supplementary Information.

### Mass constraints from *N*-body TTV modelling

**Extended Data Fig. 7:**
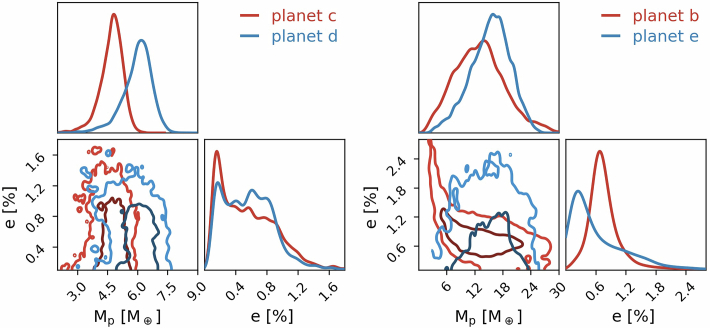
Mass and eccentricity posterior distributions. Left: joint and marginal posterior distributions of mass and eccentricity for planets c and d. Right: same as left but for planets b and e. The joint posterior panels show 1- and 2-*σ* contours for each planet.

Guided by our analytic models, our primary analysis relies on a full *N*-body dynamical model to derive the final planet parameters. We fitted the model to the observed transit times using a Bayesian framework. To be robust against outlier measurements, we adopted a log-likelihood function based on Student’s *t*-distribution^78,79^, with priors as listed in Extended Data Table 2. The posterior probability distribution was sampled using the No-U-Turn Sampler^80,81^. The model is implemented in JAX to enable automatic differentiation and is available as part of the jnkepler package^27,82^. The full mathematical details of the model implementation, the log-likelihood equation and the sampler set-up are provided in the Supplementary Information. The resulting mass and eccentricity posterior distributions are shown in Extended Data Fig. 7.

To verify the physical plausibility of our solution, we performed a detailed dynamical analysis of the posterior. We investigated both the long-term stability and the resonant state of the system using several complementary methods. First, to assess stability, we used a probabilistic classifier (SPOCK)^83^ on 1,000 samples from our posterior, which yielded a median stability probability of 95% over 10^9^ orbits. We confirmed this with direct *N*-body integrations of 128 samples for 1 Myr, which showed that the system is deeply stable and regular (minimum separation over 12*R*_H_ (mutual Hill radii), maximum semimajor axis drift of less than 0.01%, and MEGNO (Mean Exponential Growth factor of Nearby Orbits) = 2.000). As a final check, we integrated 32 posterior samples for 4 Myr, all of which were found to be stable. Second, to characterize the resonant state, our integrations show that all classical resonant angles are circulating, which we confirmed by projecting our solution onto the resonant representative plane^12^. The solution lies clearly outside the resonant island where libration would occur, confirming the non-resonant nature of the system.

### Initial thermal state and planetary evolution

**Extended Data Fig. 8:**
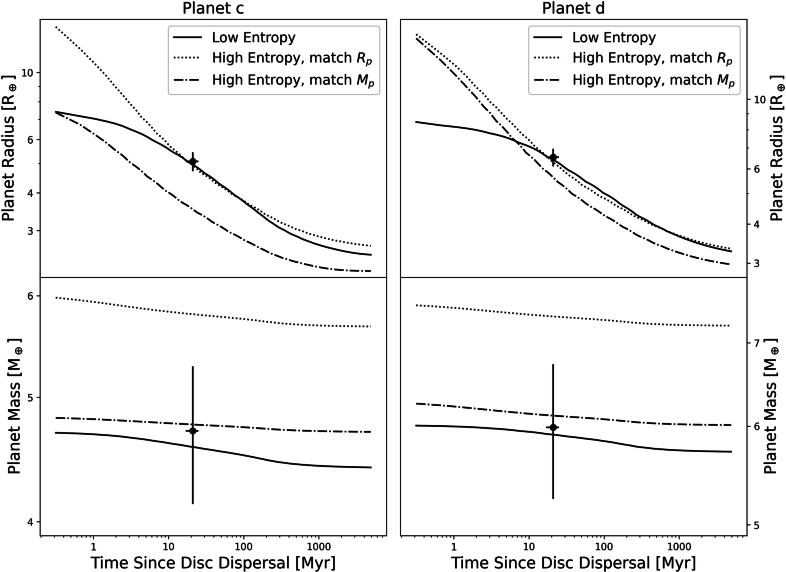
Planetary evolution models for V1298 Tau c and d. Left: plausible and implausible mass/radius evolution tracks of planet c. We ran a grid of planetary models with different masses, initial envelope fractions, and envelope entropies. The black line best matches the present-day mass and radius together, but requires low entropy. If we require high entropy (orange curve) we may match the radius, cannot match the mass. Similarly, we may match mass (blue curve), but cannot match the radius. Therefore a self-consistent formation model requires a low initial entropy. The planet evolves into a sub-Neptune of ≈2.5*R*_⊕_. Right: same as left, but for planet d, which matures into a slightly larger sub-Neptune.

Young planets with hydrogen-dominated atmospheres contract over time due to mass loss and thermal evolution. Reference ^32^ showed that young planets with measured masses and radii can be used to constrain their initial entropies. Planets with a measured mass and radius have a degeneracy between their hydrogen envelope mass fraction and their thermal state. The hydrogen envelope mass of the planet can be reduced and compensated for by an increase in its entropy. However, this can only go so far; the envelope mass cannot be reduced arbitrarily to the point where it is too small to survive mass loss. Thus, one can place a bound on the initial entropy of the planet such that it survives until today. To perform this calculation, we computed a grid of MESA evolutionary models that include photoevaporation (a comparison with ref. ^84^ indicates that these planets will be undergoing photoevaporation rather than core-powered mass loss). This model grid comprised 36 core masses, 128 initial mass fractions and 96 initial entropies. We used an identical method to that in ref. ^32^. We then compared this model grid with the observed masses and radii of the V1298 Tau planets to derive posterior distributions of the core masses, initial envelope mass fractions and initial entropies (which we encode as the initial Kelvin–Helmholtz cooling timescale of the planets). Our results indicate that all the planets had initial envelope mass fractions and core masses that are consistent with typical sub-Neptunes at billion year ages. Furthermore, the initial cooling timescales are constrained to require boil-off for planets c and d, whereas an evolution without boil-off cannot be ruled out for the outer planets. Extended Data Fig. 8 shows the models that best reproduce the present-day masses and radii of planets c and d. Interestingly, they require an initial low entropy; that is, an initial Kelvin–Helmholtz contraction time that is longer than the age of the system. Furthermore, if one considers only models with a high initial entropy, one can match the current mass or radius, but not both.

## Online content

Any methods, additional references, Nature Portfolio reporting summaries, source data, extended data, supplementary information, acknowledgements, peer review information; details of author contributions and competing interests; and statements of data and code availability are available at 10.1038/s41586-025-09840-z.

## Supplementary information

Supplementary Information A single PDF containing supplementary notes, including details about observations and TTV analyses, as well as Tables 1 and 3.

Supplementary Table 2 Transit-timing measurements. A machine-readable table (CSV format) containing the primary dataset used in this work, consisting of the measured transit times and their 1*σ* uncertainties for planets V1298 Tau b, c, d and e.

## Figures and Tables

**Extended Data Table 1:**
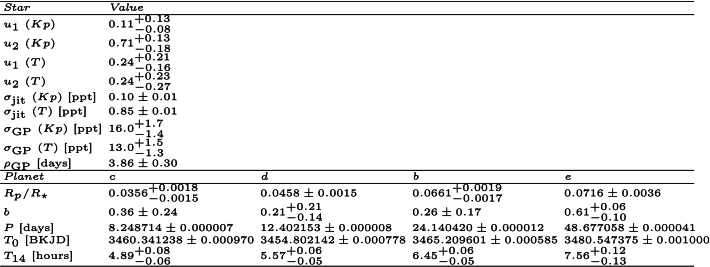
Results of *K2* -*TESS* joint modeling

**Extended Data Table 2:**
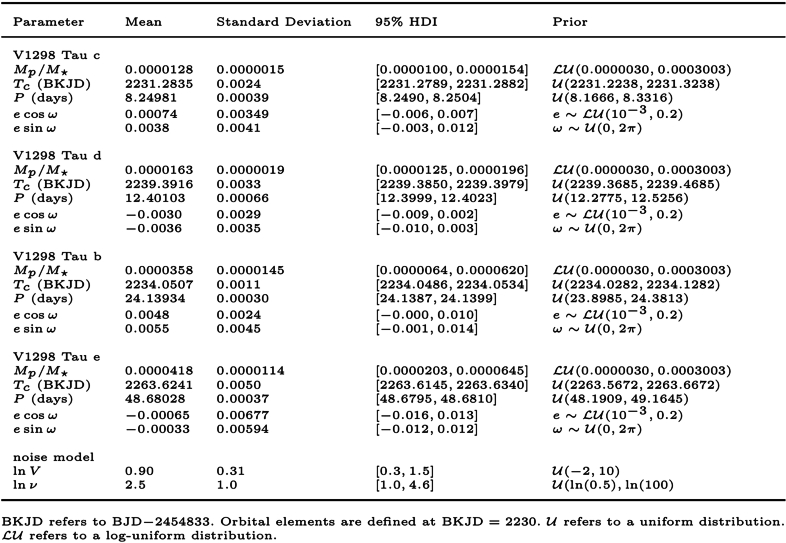
Results of *N*-body TTV modeling

## Data Availability

The transit times that form the primary dataset for this study are provided in Supplementary Table 2. K2 and TESS photometry are publicly available at MAST (https://archive.stsci.edu). Images from the Spitzer Space Telescope are available at the Spitzer Heritage Archive (https://sha.ipac.caltech.edu), and images from LCO are available at the LCO Science Archive (https://archive.lco.global). All archival data can be found by searching for the stellar identifier V1298 Tau. Photometry from Spitzer, LCO and other ground-based observations is available from the corresponding authors upon reasonable request.

## Code availability

The transit light-curve analyses were conducted using the exoplanet package available at GitHub (https://github.com/exoplanet-dev/exoplanet). The stability analysis used the SPOCK package, which is also publicly available at GitHub (https://github.com/dtamayo/spock). The *N*-body TTV analysis was performed using the publicly available jnkepler package at GitHub (https://github.com/kemasuda/jnkepler), which includes an example notebook demonstrating the analysis for this system upon publication.
