# Supplementary material for: A young progenitor for the most common planetary systems in the Galaxy
Source: Nature. 2026 Jan 7;649(8096):310–4. doi: 10.1038/s41586-025-09840-z (PMC12779570; doi:10.1038/s41586-025-09840-z)
Supplement: Supplementary file 1 — A single PDF containing supplementary notes, including details about observations and TTV analyses, as well as Tables 1 and 3. [file 41586_2025_9840_MOESM1_ESM.pdf]

nature portfolio

https://doi.org/10.1038/s41586-025-09840-z

Supplementary information

A young progenitor for the most common planetary systems in the Galaxy

In the format provided by the authors and unedited

Supplementary Information

This document contains supplementary notes and tables. The full dataset for Supplementary Table 2 is provided as a separate machine-readable file (Supplementary_Table_2.csv), while Supplementary Tables 1 and 3 are included inline.

Ground-based observations

LCO

The majority of our follow-up transit observations were obtained from 2020 to 2024 using the Las Cumbres Observatory global telescope network. We primarily used the Sinistro and MuSCAT3 instruments, mounted on the 1m and 2m telescopes, respectively, with occasional use of the SBIGSTL6303 camera on 0.4m telescopes. Exposure times ranged from 8-20 seconds across various filters (SDSS $g\prime$, $r\prime$, $i\prime$, and $z\prime_s$), with moderate defocus to improve the duty cycle. Data were calibrated using the BANZAI pipeline and processed with a custom aperture photometry pipeline. Optimal light curves were selected by minimizing flux RMS, using combinations of typically five comparison stars and aperture radii between 10 to 30 pixels. Data points beyond the saturation limit and outliers with large centroid offsets were removed.

ARCTIC

On the UT nights of 2020 January 5, January 14, February 16, and October 12, we observed transits of V1298 Tau b (first observation), and planet c (remaining observations), using the Astrophysical Research Consortium Telescope Imaging Camera on the 3.5m ARC telescope at Apache Point Observatory (APO). The ARCTIC data were obtained with the Engineered Diffuser available on the ARCTIC instrument capable of maintaining a stable point spread profile throughout the observations. All of the observations were obtained with the Semrock 867/30nm filter. The data were bias, dark and flat fielded and extracted using the `AstroImageJ` software. The October 12 observations were affected by a stellar flare during transit, which complicated the analysis, so we excluded it from our TTV analyses.

KeplerCam

On UT 2019 November 17, we observed a full transit of V1298 Tau c with the KeplerCam instrument mounted on the 1.2-meter telescope at the Fred Lawrence Whipple Observatory. KeplerCam is a single chip CCD with a Fairchild 486 detector. It has a field of view of 23.1' x 23.1' with a pixel scale of 0.672 arcsec/pixel in 2x2 binned mode. We observed the star using the SDSS z' filter with 8 second exposures. Aperture photometry was performed in `AstroImageJ` using a 10 pixel radius aperture and a sky annulus with inner and outer radii of 15 and 25 pixels, respectively.

HDI

On the nights of UT 2019 November 16 and 2020 February 16, we obtained diffuser-assisted photometry of the transits of V1298 Tau c using the Half Degree Imager,

mounted on the 0.9-meter WIYN telescope at Kitt Peak. HDI has a 4096×4096 back-illuminated CCD from e2v covering a $29.2' \times 29.2'$ Field of View at a plate scale of $0.425''$ pixel^{-1}. To obtain high precision observations of the star, we used the Engineered Diffuser installed on the 0.9m WIYN Telescope. We observed the target in the SDSS i' filter in 1x1 binning with a gain of 1.3 e$^-$ / ADU, using an exposure time of 45s. To extract the photometry, we explored using a number of different apertures in `AstroImageJ`, where we ultimately adopted a reduction using a 18-20 pixel ($7.6-8.5''$) radius aperture and a sky annulus with inner and outer radii of 25 pixels ($11''$) and 40-50 pixels ($17-21''$), respectively, which resulted in the lowest overall photometric scatter.

TMMT

On the nights of UT 2019 November 8, November 16, and December 11, we observed transits of V1298 Tau c using the Three-hundred MilliMeter Telescope at Las Campanas Observatory. The observations were performed slightly out of focus in the Cousins I_C filter, resulting in a point spread function FWHM of $6-7''$. TMMT has a gain of 1.4 e$^-$ / ADU and a plate scale $1.2''$ pixel^{-1} in the 1×1 binning mode used. For all observations, we used an exposure time of 30s. To extract the photometry, we explored using a number of different apertures in `AstroImageJ`, where we ultimately adopted a reduction using a 7-8 pixel ($8.4-9.6''$) radius aperture and a sky annulus with inner and outer radii of 10 ($12''$) and 18-20 pixels ($22-24''$), respectively, which resulted in the lowest overall photometric scatter.

MuSCAT

On the night of 2020 October 20 UT, we observed V1298 Tau with MuSCAT, mounted on the NAOJ 1.9m telescope located in Okayama, Japan. Data were calibrated using the standard instrument pipeline, including dark subtraction, flat fielding, and linearity correction. As was done for LCO data, light curves were produced using a custom aperture photometry pipeline.

MuSCAT2

On the nights of 2022 Novemeber 3, 2023 October 5, 2023 November 7, 2023 December 2, 2023 December 10, and 2024 January 4, we observed V1298 Tau with MuSCAT2, mounted on the 1.5m TCS telescope (Telescopio Carlos Sanchez) at Teide Observatory, Tenerife. MuSCAT2 data calibration and photometry were performed in the same fashion as the MuSCAT data described above.

Koyama

On 2020 December 9 UT, we observed a partial transit of V1298 Tau d using the ADLER camera mounted on the 1.3m Araki Telescope telescope at Koyama Astronomical Observatory, Kyoto Sangyo University, Japan. ADLER has a Spectral Instruments 850 series 2048×2048-pixel CCD with a pixel scale of 0.357 $''$/pix, yielding a $12' \times 12'$ FoV. A $\sim$4.7-hour sequence of 120 second exposures was obtained in

$z\prime$-band from 08:39 to 13:18 UT, using moderate telescope defocus to avoid saturation of the target and comparison stars. On 2023 March 8 from 10:09 to 12:54 UT, and again on 2024 February 9 from 10:11 to 13:04 UT, we observed partial transits of V1298 Tau b using the same instrumental setup.

Datasets containing flares

We modeled datasets containing flares using our standard approach, augmented with a parametric flare model that characterizes flares by their peak time ($t_{\rm peak}$), amplitude (a), and full-width at half-maximum (w). Significant flares were observed in ARCTIC data (2020 October 12), KeplerCam data (2023 September 24), and LCO data (2023 December 18), with amplitudes ranging from 6 to 42 ppt and timescales of 14 to 21 minutes. These datasets and models are shown in Extended Data Fig. 5. The ARCTIC dataset yielded a flare with parameters $t_{\rm peak} = 2459134.7691 \pm 0.0003$ BJD, $a = 6.0 \pm 0.5$ ppt, and $w = 14.3 \pm 2.2$ minutes; the KeplerCam dataset yielded $t_{\rm peak} = 2460211.8506 \pm 0.0002$ BJD, $a = 42.0 \pm 1.2$ ppt, and $w = 21.1 \pm 1.4$ minutes; the LCO dataset yielded 2460296.8921 ± 0.0002 BJD, $a = 30.1 \pm 1.0$ ppt, and $w = 18.1 \pm 1.1$ minutes. These flare observations may prove valuable for future studies of the activity of V1298 Tau.

Mass constraints from analytic TTV modeling

The four planets in the V1298 Tau system tug on one another due to gravity. These interactions result in transit-timing variations (TTVs) of several hours. Analytic models of TTVs have been developed in numerous previous works, and several groups have developed N-body codes. Many TTV studies have been performed for planets from the four-year *Kepler* mission. These studies had the benefit of near-continuous sampling over a full TTV period. The V1298 Tau dataset presents a different set of challenges due to low phase coverage and heterogeneous uncertainties. We thus analyzed the system using a mix of analytic and numerical models.

Lithwick et al. (2012; hereafter L12) developed an analytic model for TTVs due to pairs of planets near (but not too close to or in) resonance. Subsequently, Nesvorny et al. (2016; hereafter N16) developed a more general analytic model valid for planets both in and near resonance. The L12 model may be derived as a limiting case of the N16 model. We used both models, where appropriate.

L12 showed that pairs of planets near resonance exhibit anti-correlated and sinusoidal TTVs. The TTV period (sometimes called the 'super-period') is given by

$$P_{12} = \frac{P_2}{j|\Delta|} \tag{1}$$

where j defines the first-order resonance and Δ characterizes the proximity to it:

$$\Delta = \frac{P_2}{P_1}\frac{j-1}{j} - 1. \tag{2}$$

We computed the proximity parameter and TTV period using a linear fit the measured transit times. We found $\Delta_{\rm cd} = 0.23\%$, $\Delta_{\rm db} = -2.67\%$, and $\Delta_{\rm be} = 0.82\%$. The expected TTV periods are $P_{\rm cd} =$ 1834 days, $P_{\rm db} =$ 451 days, and $P_{\rm be} =$ 2953 days.

The TTV amplitude is proportional to $1/\Delta$. Since, $\Delta_{\rm db}$ is much larger than both $\Delta_{\rm cd}$ and $\Delta_{\rm be}$ the TTVs of c and d will be dominated by c-d interactions and the TTVs of b and e will be dominated by b-e interactions. The b-d interactions will be second order corrections. Having thus separated the problem, we fit the following multi-harmonic model to the TTV timeseries.

$$t_{\rm c} = t_{\rm c,0} + P_c i_{\rm c} + A_{\rm cd} \sin(2\pi/P_{\rm cd}(t - t_{\rm cd,0})) \tag{3}$$

$$t_{\rm d} = t_{\rm d,0} + P_d i_{\rm d} + A_{\rm dc} \sin(2\pi/P_{\rm cd}(t - t_{\rm cd,0})) \tag{4}$$

$$t_{\rm b} = t_{\rm b,0} + P_b i_{\rm b} + A_{\rm be} \sin(2\pi/P_{\rm be}(t - t_{\rm be,0})) \tag{5}$$

$$t_{\rm e} = t_{\rm e,0} + P_e i_{\rm e} + A_{\rm eb} \sin(2\pi/P_{\rm be}(t - t_{\rm be,0})) \tag{6}$$

For each planet, a baseline linear ephemeris is specified by the first two terms, where i is an integer that indexes each transit. Sinusoidal TTVs are specified by the last term. The parameters $A_{\rm cd}$, $P_{\rm cd}$, and $t_{\rm cd,0}$ specify the amplitude, period, and phase of the TTVs of planet c due to d. Equivalent parameters for other planets follow the same convention. Note that due to the anti-correlated nature of TTVs, we set $P_{\rm cd} = P_{\rm db}$, $t_{\rm cd,0} = t_{\rm dc,0}$. An equivalent symmetry exists for the b-e pair. We modeled the transit times allowing the following 16 parameters to vary: $\{t_{\rm c,0}$, $P_{\rm c}$, $A_{\rm cd}$, $P_{\rm cd}$, $t_{\rm cd}$, $t_{\rm d,0}$, $P_{\rm d}$, $A_{\rm dc}$, $t_{\rm b,0}$, $P_{\rm b}$, $A_{\rm be}$, $P_{\rm be}$, $t_{\rm be}$, $t_{\rm e,0}$, $P_{\rm e}$, $A_{\rm eb}$ $\}$. We explored credible models using MCMC. Extended Data Fig. 6 shows the measured transit times and draws from the set of credible models.

We found $P_{\rm be}$ to be 2852 ± 50 days. The b-e pair is well described by a sinusoid with a period matching the L12 prediction of 2953 days, indicating that the L12 model is an adequate model of the TTV signal. In contrast, $P_{\rm cd}$ is 1604 ± 12 days, which is inconsistent with the L12 predicted value of 1834 days at high significance. The L12 model is not appropriate for the c-d pair and the more general N16 model is needed.

TTV analysis of planets c and d

Observed TTVs depend on variations in the position of the planet in its orbit and the orbit's orientation according to:

$$\delta t = \frac{1}{n}(-\delta\lambda + 2\delta h) \tag{7}$$

where n is the mean motion, λ is the mean longitude, $h = e \sin \varpi$, and ϖ is the longitude of periastron. When a system is sufficiently far from resonance, sinusoidal variations due to λ and h have the same period, $P_{\rm ttv,\lambda} \approx P_{\rm ttv,h}$. Sufficiently close to resonance, the two frequencies diverge and $P_\lambda < P_h$.

N16 showed that λ oscillates with a period

$$P_{\rm ttv,\lambda} = \frac{P_1 + P_2}{2}\frac{P_\tau}{2\pi}\left(\frac{m_1 + m_2}{M_\star}\right)^{-2/3}\left[\frac{3}{2}k(k-1)|f_1|f_2\right]^{-1/3} \tag{8}$$

where $k = 3$ is the degree of resonance, $f_1 = -2.025$ and $f_2 = 2.484$ are order unity resonant coefficients of the disturbing function, and P_τ is the dimensionless period of resonant libations (typically $\approx 2-4$). Thus, P_λ is sensitive to the sum of the planet masses. In addition, the TTV amplitudes are

$$A_1 = \frac{3(k-1)}{\Lambda_1 \nu} \frac{P_\tau}{2\pi} A_\Psi \tag{9}$$

$$A_2 = \frac{3k}{\Lambda_2 \nu} \frac{P_\tau}{2\pi} A_\Psi \tag{10}$$

where $\nu = (3/2)[(k-1)^2 n_1/\Lambda_1 + k^2 n_2/\Lambda_2]$, $\Lambda_j = m_j \sqrt{GM_\star a_j}$, and A_Ψ is the dimensionless amplitude of resonant librations. We do not know A_Ψ in advance, which could range from 0 for an exact resonance to >1 for large librations. However, we note that $A_1/A_2 \simeq -[(k-1)/k]^{2/3} M_2/M_1$, and thus the ratio of TTV amplitudes is closely related to the planet mass ratio.

The TTV period and amplitudes together constrain both the mass ratio and the sum of the masses, which together are sufficient to constrain the masses of the individual planets. We did so through a simple, albeit brute-force, importance sampling scheme. We simulated 10^{10} planet pairs with properties drawn according to the following distributions:

$$m_1 \sim \text{LogUniform}(10^{-1}, 10^2)\ M_\oplus \tag{11}$$

$$M_2/M_1 \sim \text{LogUniform}(0.5, 2) \tag{12}$$

$$e_1 \sim e_2 \sim \text{LogUniform}(10^{-4}, 10^{-1}) \tag{13}$$

$$\sigma_1 = k\lambda_2 - (k-1)\lambda_1 - \varpi_1 \sim U(0, 2\pi) \tag{14}$$

$$\sigma_2 = k\lambda_2 - (k-1)\lambda_1 - \varpi_2 \sim U(0, 2\pi) \tag{15}$$

$$\tag{16}$$

We then applied the formulae in N16 to determine $A_{\rm cd,sim}$, $A_{\rm dc,sim}$, $P_{\rm cd,sim}$ for each simulated planet pair. We then computed

$$\chi^2 = \left(\frac{A_{\rm cd,sim} - A_{\rm cd}}{\sigma(A_{\rm cd})}\right)^2 + \left(\frac{A_{\rm dc,sim} - A_{\rm dc}}{\sigma(A_{\rm dc})}\right)^2 + \left(\frac{P_{\rm cd,sim} - P_{\rm cd}}{\sigma(P_{\rm cd})}\right)^2 \tag{17}$$

to quantify the degree to which each simulated system matched the observed system. Because the prior volume is large, and $\{A_{\rm cd}, A_{\rm dc}, P_{\rm cd}\}$ have small fractional uncertainties, our sampling efficiency is low. The fraction of planet pairs with $\chi^2 < 10$ was $\sim 10^{-4}$. The likelihood of any given model given the data is $\mathcal{L} \propto e^{-\chi^2/2}$. We therefore weighted each sample by $\mathcal{L}$ to derive credible intervals for each parameter. We found $m_c = 2.7^{+1.7}_{-0.8}\ M_\oplus$. Not surprisingly, the mass ratios were measured with high precision $m_d/m_c = 1.18 \pm 0.02$. Thus, $m_d = 3.2^{+2.1}_{-1.0}\ M_\oplus$. Finally, we determined that eccentricities were no more than a few percent: $e_c < 0.04$ and $e_d < 0.03$ at 95% confidence.

Let us pause to reflect on the remarkable insights achieved from the measurement of just three quantities. We have determined that the inner two planets are only a few Earth masses, despite being ≈ 6 Earth-radii and have eccentricities of only a few percent. These analytic results serve as a sanity check on our adopted N-body model.

TTV analysis of planets b and e

As stated previously, the b-e pair may be treated with the L12 formalism, which predicts anti-correlated, sinusoidal TTVs with a period and amplitudes given by:

$$P_{\rm ttv} = \frac{P_2}{j|\Delta|}, \tag{18}$$

$$A_1 = P_1 \frac{\mu_2}{\pi k^{2/3}(j-1)^{1/3}\Delta}\left(-f - \frac{3}{2}\frac{Z^*_{\rm free}}{\Delta}\right) \tag{19}$$

$$A_2 = P_2 \frac{\mu_1}{\pi k \Delta}\left(-g + \frac{3}{2}\frac{Z^*_{\rm free}}{\Delta}\right), \tag{20}$$

respectively, where μ is the planet-star mass ratio, and f and g are order unity scalar coefficients that depend on j and Δ and are given in L12. For the b-e pair, $f = -1.190$ and $g = 0.4284$, neglecting terms of $\mathcal{O}(\Delta)$. $Z^*_{\rm free}$ is the complex conjugate of the following linear combination of the planets complex eccentricities:

$$Z_{\rm free} = f z_{\rm free,1} + g z_{\rm free,2}, \tag{21}$$

where

$$z = e\cos\varpi + ie\sin\varpi. \tag{22}$$

We note a few differences between the L12 and N16 TTV models. In the L12 limit, the TTV period does not depend on the planet masses, only the orbital periods; in contrast, in the N16 model it depends on the sum of the masses. The individual TTV amplitudes depend on the product of planet mass times a linear combination of the eccentricities. Thus, we anticipate good constraints on M_2/M_1 and the mass-eccentricity product, but not on individual masses or eccentricities. This 'mass-eccentricity degeneracy' has been discussed extensively in the literature, e.g. Deck et al. (2015) and Hadden et al. (2016). Also in the L12 formalism, the TTV amplitude is treated as a complex value, where the magnitude encodes the amplitude of the signal and the phase encodes the shift of the TTV signal relative to the time when the line-of-conjunctions points at the observer.

The amplitudes $|A_1|$ and $|A_2|$ are given as A_{be} and A_{eb} in Supplementary Table 3. We observe a small phase shift $\phi = -0.116 \pm 0.017$ radians which indicates the eccentricities are non-zero $|Z_{\rm free}| > 0$. However, since the phase shift is small compared to unity, it is unlikely that $|Z_{\rm free}| \gg \Delta$. Therefore, $0 < |Z_{\rm free}| \lesssim \Delta = 0.82$; thus, the individual eccentricities are a few percent or less.

As in the previous section, we used importance sampling to measure the planet masses. We simulated 10^8 planet pairs with properties drawn according to the following distributions:

$$\mu_1 \sim \text{LogUniform}(10^0, 10^2)\ M_\oplus/M_\odot \tag{23}$$
$$M_2/M_1 \sim \text{LogUniform}(0.2, 5) \tag{24}$$
$$|Z_{\text{free}}| \sim \text{LogUniform}(10^{-4}, 10^{-1}) \tag{25}$$
$$\text{phase}(Z_{\text{free}}) \sim \text{Uniform}(0, 2\pi) \tag{26}$$

We then applied the formulae in L12 to determine $A_{\text{be,sim}}$, $A_{\text{eb,sim}}$, and ϕ_{be} for each simulated planet pair. We then computed

$$\chi^2 = \left(\frac{A_{\text{be,sim}} - A_{\text{be}}}{\sigma(A_{\text{be}})}\right)^2 + \left(\frac{A_{\text{eb,sim}} - A_{\text{eb}}}{\sigma(A_{\text{eb}})}\right)^2 + \left(\frac{\phi_{\text{be,sim}} - \phi_{\text{be}}}{\sigma(\phi_{\text{be}})}\right)^2 \tag{27}$$

The samples were weighted following the procedure in the previous section. We found $M_b = 31^{+14}_{-17}\ M_\oplus$, $M_e = 24^{+4}_{-8}\ M_\oplus$, and $\log|Z_{\text{free}}| = -3.0^{+0.6}_{-0.2}$. The large fractional mass uncertainties stem from the mass-eccentricity degeneracy discussed earlier. One may decrease the mass of planets b and e (keeping their ratio fixed) and increase Z_{free} and produce the same TTV curve. Increased phase coverage of planet e's TTV curve will not dramatically improve the mass measurements. However, there are a number of possible avenues to improve these measurements. As shown in Fig. 1 of the main article, L12 interactions between planet d and b lead to a low-amplitude $\sim$500 day TTV period. Better constraining this signal will improve M_d/M_b and hence M_b and M_e. Secondary eclipse times directly measure $e\cos\omega$ (Winn 2010) and break the mass-eccentricity degeneracy. Exceptionally precise timing measurements of consecutive transits could resolve synodic chopping which does not suffer from the mass-eccentricity degeneracy (Deck et al., 2015).

Mass constraints from N-body TTV modeling

Our N-body model computes transit times of each planet considering gravitational interactions between the planets and the star. The interaction was assumed to be Newtonian, and the light travel time was ignored. The model parameters are four planets' masses relative to that of the host star, and the osculating orbital elements (period P, eccentricity e, argument of periastron ω, and time of inferior conjunction T_c) defined at the start of integration, BKJD = 2230, where T_c is converted to the time of periastron passage τ via $2\pi(T_c - \tau)/P = E_0 - e\sin E_0$ with $E_0 = 2\arctan\left[\sqrt{\frac{1-e}{1+e}}\tan\left(\frac{\pi}{4} - \frac{\omega}{2}\right)\right]$. We assume coplanar orbits, and fix the orbital inclinations to be $\pi/2$ and longitudes of the ascending node to be 0 for all the planets. In defining the orbital elements, we choose the sky plane to be the reference plane, and adopt the $+Z$-axis pointing toward the observer.

The masses and osculating orbital elements are converted to Jacobi coordinates, using the total interior mass as the mass entering in the conversion from periods to coordinates. An N-body integration is performed using a symplectic integrator with a fixed time step of 0.3 days (i.e., $\approx 1/27$ of the innermost period), which results in

a typical fractional energy error of $\mathcal{O}(10^{-8})$. The resulting orbits are then used to derive transit times of the planets following a standard iterative scheme, in which a fourth-order Hermite integrator is used. The N-body code is implemented in `JAX` to enable automatic differentiation with respect to the input orbital elements and mass ratios, and is available through GitHub as a part of the `jnkepler` package.

When sampling from the posterior probability distribution, we adopt the log-likelihood function given by the Student's t distribution:

$$\ln \mathcal{L}(\theta) = \sum_i \left\{ -\frac{\nu+1}{2} \ln \left[1 + \frac{(t_i - m_i(\theta))^2}{\nu V \sigma_i^2} \right] - \frac{1}{2} \ln(\pi \nu V \sigma_i^2) + \ln \Gamma\left(\frac{\nu+1}{2}\right) - \ln \Gamma\left(\frac{\nu}{2}\right) \right\}, \tag{28}$$

where $m_i(\theta)$ is the model, ν is the number of degrees of freedom, V is the scale of the distribution, and $\Gamma(x)$ is the Gamma function. The prior is assumed to be separable for each parameter and is listed in Extended Data Table 2. In practice, the sampling was performed assuming a uniform prior distribution for e, i.e. the results were calculated from posterior samples that were resampled with weights proportional to $1/e$. The sampling was performed using the No-U-Turn Sampler as implemented in `NumPyro`. We ran four chains in parallel for 2,000 steps. The resulting chains had a split Gelman-Rubin statistic of $\hat{R} < 1.02$, and the estimated number of effective samples was at least 500 for each parameter. The mass and eccentricity posterior distributions are shown in Extended Data Fig. 7.

Table 1 Summary of transit observations analyzed in this work.

Date Start (UT)	Date End (UT)	Planet	Instrument	Filter	Exposure Time (s)
2015-02-08 07:13	2015-04-20 04:17	b,c,d,e	*Kepler*/*K2*	*Kepler* bandpass	1800
2019-06-01 05:59	2019-06-01 17:31	b	*Spitzer*	IRAC2	2
2019-11-09 02:56	2019-11-09 06:31	c	TMMT	Cousins I	30
2019-11-17 04:13	2019-11-17 08:05	c	TMMT	Cousins I	30
2019-11-17 04:39	2019-11-17 10:28	c	HDI/WIYN0.9m	SDSS i'	45
2019-11-17 05:25	2019-11-17 12:08	c	KeplerCam	SDSS z'	8
2019-12-12 00:42	2019-12-12 00:42	c	TMMT	Cousins I	30
2019-12-28 07:03	2019-12-28 21:03	c,d	*Spitzer*	IRAC2	2
2020-01-04 17:42	2020-01-05 06:24	b	*Spitzer*	IRAC1	0.4
2020-01-05 01:45	2020-01-05 07:04	b	ARCTIC/ARC3.5m	Semrock 857/30	30
2020-01-14 02:04	2020-01-14 07:07	c	ARCTIC/ARC3.5m	Semrock 857/30	30
2020-02-16 01:44	2020-02-16 05:58	c	ARCTIC/ARC3.5m	Semrock 857/30	30
2020-02-16 02:23	2020-02-16 06:22	c	HDI/WIYN0.9m	SDSS i'	45
2020-10-08 14:14	2020-10-08 18:10	d	LCO1m/Sinistro@SSO	z_s	30
2020-10-12 04:28	2020-10-12 11:40	c	ARCTIC/ARC3.5m	Semrock 857/30	30
2020-10-20 12:47	2020-10-20 20:29	b,c	Okayama1.9m/MuSCAT	g',r',z'_s	60,40,60
2020-12-09 08:39	2020-12-09 13:18	d	Koyama1.3m/ADLER	z_s	120
2021-09-16 16:04	2021-11-05 22:41	b,c,d,e	*TESS*	*TESS* bandpass	120
2022-10-07 23:38	2022-10-08 06:15	c	LCO1m/Sinistro@TO	r'	15
2022-10-18 11:55	2022-10-18 15:29	e	LCO40cm/SBIG@HO	Johnson-Cousins V	12
2022-10-18 13:08	2022-10-18 15:35	e	LCO2m/MuSCAT3@HO	g',r',i',z'_s	8,8,12,20
2022-11-03 23:03	2022-11-04 05:06	d	TCS/MuSCAT2@TO	g',r',i',z'_s	20,15,20,20
2022-11-07 23:41	2022-11-08 03:55	b	LCO40cm/SBIG@TO	r'	90
2022-11-16 08:00	2022-11-16 09:25	d	LCO2m/MuSCAT3@HO	g',r',i',z'_s	8,8,12,20
2022-11-28 21:59	2022-11-29 02:17	d	LCO1m/Sinistro@TO	r'	15
2022-11-28 23:03	2022-11-29 03:42	d	TCS/MuSCAT2@TO	g',r',i',z'_s	8,8,5,5
2022-12-02 03:40	2022-12-02 07:14	b	LCO1m/Sinistro@CTIO	r'	15
2022-12-02 03:54	2022-12-02 06:52	b	LCO1m/Sinistro@MO	r'	15
2022-12-02 04:12	2022-12-02 07:04	b	LCO1m/Sinistro@MO	r'	15
2022-12-26 00:59	2022-12-26 04:50	b	LCO1m/Sinistro@CTIO	r'	15
2023-01-05 03:09	2023-01-05 08:05	d	LCO1m/Sinistro@MO	r'	15
2023-01-05 03:38	2023-01-05 07:34	d	LCO1m/Sinistro@MO	r'	15
2023-01-15 00:54	2023-01-15 03:04	c	LCO1m/Sinistro@CTIO	r'	15
2023-01-15 00:54	2023-01-15 03:33	c	LCO1m/Sinistro@CTIO	r'	15
2023-01-15 01:20	2023-01-15 08:03	c	LCO1m/Sinistro@MO	r'	15
2023-01-23 21:08	2023-01-24 01:02	e	LCO1m/Sinistro@TO	r'	15
2023-01-23 22:08	2023-01-24 01:01	e	LCO1m/Sinistro@TO	r'	15
2023-02-11 01:38	2023-02-11 04:25	d	LCO1m/Sinistro@MO	r'	20
2023-02-11 01:38	2023-02-11 06:00	d	LCO1m/Sinistro@MO	r'	20
2023-03-08 10:09	2023-03-08 12:54	b	Koyama1.3m/ADLER	z_s	120
2023-07-31 13:16	2023-07-31 15:02	b	LCO2m/MuSCAT3@HO	g',r',i',z'_s	8,8,12,20
2023-08-24 17:05	2023-08-24 19:13	b	LCO1m/Sinistro@CTIO	r'	20
2023-08-24 17:05	2023-08-24 19:13	b	LCO1m/Sinistro@CTIO	r'	20
2023-10-06 01:07	2023-10-06 06:05	c	TCS/MuSCAT2@TO	g',r',i',z'_s	10,12,12,15
2023-10-06 01:08	2023-10-06 04:03	c	LCO1m/Sinistro@TO	r'	20
2023-10-06 05:52	2023-10-06 07:36	c	LCO1m/Sinistro@CTIO	r'	20
2023-10-06 05:57	2023-10-06 07:36	c	LCO1m/Sinistro@CTIO	r'	20
2023-10-06 06:08	2023-10-06 07:36	c	LCO1m/Sinistro@CTIO	r'	20
2023-10-14 06:53	2023-10-14 09:19	c	LCO1m/Sinistro@MO	r'	20
2023-10-14 07:38	2023-10-14 09:34	c	LCO1m/Sinistro@MO	r'	20
2023-10-17 01:53	2023-10-17 03:26	d	LCO1m/Sinistro@TO	r'	20
2023-10-17 06:53	2023-10-17 09:50	d	LCO1m/Sinistro@MO	r'	20
2023-10-17 07:08	2023-10-17 09:34	d	LCO1m/Sinistro@MO	r'	20
2023-10-22 13:09	2023-10-22 15:35	c	LCO2m/MuSCAT3@HO	g',r',i',z'_s	8,8,12,20
2023-11-08 00:34	2023-11-08 05:52	c	TCS/MuSCAT2@TO	g',r',i',z'_s	5,5,5,5
2023-11-11 23:40	2023-11-12 02:16	e	LCO1m/Sinistro@SAAO	r'	20
2023-11-11 23:50	2023-11-12 02:15	e	LCO1m/Sinistro@TO	r'	20
2023-11-11 07:20	2023-11-12 09:46	e	LCO2m/MuSCAT3@HO	g',r',i',z'_s	8,8,12,20
2023-11-16 07:42	2023-11-16 10:58	c	LCO2m/MuSCAT3@HO	g',r',i',z'_s	8,8,12,20
2023-11-16 12:26	2023-11-16 14:00	c	LCO2m/MuSCAT3@HO	g',r',i',z'_s	8,8,12,20
2023-11-23 12:40	2023-11-23 14:05	d	LCO2m/MuSCAT3@HO	g',r',i',z'_s	8,8,12,20
2023-12-02 22:34	2023-12-03 03:36	c	TCS/MuSCAT2@TO	g',r',i',z'_s	11,12,11,11
2023-12-11 01:43	2023-12-11 04:03	c	TCS/MuSCAT2@TO	g',r',i',z'_s	7,7,4,4
2023-12-11 02:10	2023-12-11 10:30	c	KeplerCam	SDSS z'	30
2023-12-18 01:44	2023-12-18 07:26	d	KeplerCam	SDSS z'	30
2023-12-23 02:54	2023-12-23 05:50	b	LCO1m/Sinistro@MO	r'	20
2023-12-23 02:55	2023-12-23 06:50	b	LCO1m/Sinistro@MO	r'	20
2023-12-30 22:39	2023-12-31 02:05	e	LCO1m/Sinistro@TO	r'	20
2024-01-04 19:49	2024-01-04 22:52	c	TCS/MuSCAT2@TO	g',r',i',z'_s	8,8,7,8
2024-01-13 01:22	2024-01-13 04:49	c	LCO1m/Sinistro@TO	r'	20
2024-01-24 06:37	2024-01-24 10:17	d	LCO2m/MuSCAT3@HO	g',r',i',z'_s	8,8,12,20
2024-02-09 10:11	2024-02-09 13:04	b	Koyama1.3m/ADLER	z_s	120
2024-02-23 07:18	2024-02-23 08:58	c	LCO2m/MuSCAT3@HO	g',r',i',z'_s	8,8,12,20
2024-03-04 20:00	2024-03-04 21:24	b	LCO1m/Sinistro@TO	r'	20

CTIO = Cerro Tololo Inter-American Observatory in District IV, Chile
HO = Haleakala Observatory on Maui, Hawaii
MO = McDonald Observatory at Fort Davis, Texas
SSO = Siding Spring Observatory in New South Wales, Australia
TO = Teide Observatory on Tenerife in the Canary Islands, Spain

Table 3 Posteriors from the multi-harmonic fit.

Parameter	Value	Unit
$P_{\rm c}$	8.249164 ± 0.000003	days
$P_{\rm d}$	12.401394 ± 0.000009	days
$P_{\rm b}$	24.140006 ± 0.000017	days
$P_{\rm e}$	48.677714 ± 0.000053	days
$t_{\rm c,0}$	2231.218 ± 0.002	BKJD
$t_{\rm d,0}$	2239.466 ± 0.002	BKJD
$t_{\rm b,0}$	2234.093 ± 0.002	BKJD
$t_{\rm e,0}$	2263.587 ± 0.003	BKJD
$A_{\rm cd}$	0.066 ± 0.001	days
$A_{\rm dc}$	-0.073 ± 0.001	days
$A_{\rm be}$	0.0491 ± 0.0005	days
$A_{\rm eb}$	-0.040 ± 0.002	days
$P_{\rm cd}$	1604 ± 12	days
$P_{\rm be}$	2852 ± 50	days
